# Amino acid and energy digestibility of conventional corn dried distillers grains with solubles, high protein distillers dried grains, and corn fermented protein, and impacts on turkey poult growth performance and intestinal characteristics

**DOI:** 10.1016/j.psj.2024.104082

**Published:** 2024-07-10

**Authors:** Zoie K. VerBeek, Claudia DeLeon, Brian J. Kerr, Gerald C. Shurson, Sally L. Noll, Dawn A. Koltes

**Affiliations:** ⁎ Department of Animal Science, Iowa State University, Ames, IA 50011, USA; † USDA-ARS-National Laboratory for Agriculture and the Environment, Ames, IA, 50011, USA; ‡ Department of Animal Science, University of Minnesota, St. Paul, MN, 55108, USA

**Keywords:** amino acid digestibility, corn fermented protein, distiller dried grain with soluble, energy digestibility, high protein distillers dried grain

## Abstract

Amino acids (**AA**) are an expensive nutritional components of poultry diets. Distillers dried grains with solubles (**DDGS**) is the primary co-product produced by the dry grind bioethanol industry, although new technologies are being implemented to produce high protein distillers dried grains (**HP-DDG**) and corn fermented protein (**CFP**), but data on their nutritive value in poultry are lacking. Two experiments (**EXP**) were conducted to determine the energy and AA digestibility of DDGS, HP-DDG, and CFP in poults in addition to a feeding trial to evaluate increasing dietary levels of HP-DDG and CFP on growth performance and intestinal characteristics. In EXP 1, 6 different DDGS sources were evaluated using poults to determine their nitrogen-corrected apparent metabolizable energy (**AME_n_**) concentrations, and cecectomized roosters were used to determine their standardized ileal (**SID**) AA digestibility (**SID-AA**). In EXP 2, AME_n_ and SID-AA for HP-DDG and CFP were determined in young poults, and a feeding trial was conducted to evaluate growth performance and intestinal morphology and permeability of poults fed diets containing 7.5 and 15% HP-DDG or CFP from 1 to 32 d of age. In EXP 1, the AME_n_ concentration among the DDGS samples ranged from 2,530 to 3,573 kcal/kg DM but was not different (*P* = 0.57) among the samples, with an average SID for LYS of 66.6%. In EXP 2, different (*P* = 0.001) AME_n_ concentrations for HP-DDG and CFP were observed (3,114 and 3,760 kcal/kg DM, respectively), with the SID for LYS being 66.55 and 77.00% for HP-DDG and CFP, respectively. Including HP-DDG or CFP into the diet at 7.5 and 15% had no effect (*P* > 0.05) on growth, feed intake, or feed conversion. Neither co-product nor its inclusion rate affected intestinal morphology and permeability (*P* > 0.05). Overall, DDGS, HP-DDG, and CFP are excellent sources of AME_n_ and digestible AA, with dietary inclusion rates of up to 15% of HP-DDG or CFP having no impact on growth or intestinal characteristics.

## INTRODUCTION

Feed accounts for approximately two-thirds of the cost to raise a turkey (US Poultry Industry Manual, 2022) with most expensive dietary components being energy, amino acids (**AA**),and phosphorus. Distillers dried grains with solubles (**DDGS**) is a co-product from the corn dry grind process used in the bioethanol industry (Iram et al., 2020; Pont et al., 2023) and is an excellent source of digestible AA, energy, and phosphorus (Batal and Dale, 2006; Rochell et al., 2011; Corray et al., 2019).

The removal of a portion of the oil in the manufacture of corn derived DDGS has become common place, but as oil in DDGS is removed, both nutritive and non-nutritive components are concentrated in the DDGS, including protein and fiber, respectively (Martinez-Amezcua et al., 2007; Meloche et al., 2013; 2014). In addition, there are also many other co-products derived from the corn milling and ethanol industry. This is due to processing differences resulting in corn co-products with different nutritional profiles and feeding applications (Shurson, 2023), products which typically have less fiber (Urriola et al., 2010) and more AA (Adeola and Ragland, 2016) compared with conventional DDGS. Two corn co-products from the dry grind bioethanol industry, high protein distillers dried grains (**HP-DDG**) and corn fermented protein (**CFP**), are of interest in the poultry industry due to their lower fiber and greater AA concentrations. Even though DDGS are used extensively in swine and broiler production (Stein and Shurson, 2009; Adeola and Zhai, 2012), data for the nutritive and feeding value of DDGS, HP-DDG, or CFP in turkey production are limited.

In addition to differences in energy and AA concentrations, many corn co-products contain elevated levels of dietary fiber (e.g., non-starch polysaccharides, **NSP**). Poultry digest NSP poorly, and due to excess fiber in the diet, the overall digestibility of the diet is reduced (de Vries et al., 2012; Bederska-Łojewska et al., 2017; Nguyen et al., 2022) and intestinal permeability may be increased (Tellez et al., 2014; Pont et al., 2023). Even though research has shown that feeding HP-DDGS to broilers had no impact on intestinal health (Mustafa et al., 2017), the impact of feeding DDGS, HP-DDG, or CFP on intestinal health in turkeys is unknown. Therefore, the objectives of this study were to determine the metabolizable energy and AA digestibility in 6 conventional DDGS samples as well as higher protein HP-DDG and CFP sources. Although a previous growth performance study to evaluate feeding DDGS to turkeys has been conducted (Świątkiewicz and Koreleski, 2008), our study determined the impact of feeding increasing dietary inclusion rates of HP-DDG and CFP on poult performance and intestinal health.

## MATERIALS AND METHODS

### Animal Care

All experimental procedures were approved by the Institutional Animal Care and Use Committees at the University of Minnesota (IACUC # 1008A87113, 1201B69531, 1207B16841) and University of Illinois (#23092) for Experiment 1 and at Iowa State University (IACUC #21-268) for Experiment 2.

### Experiment 1

For Experiment 1 (**EXP 1**), 6 DDGS samples reflective of different nutritional composition were obtained from the dry grind bioethanol industry (Table 1), and were the same samples used in previous studies with broilers (Meloche et al., 2014) and swine (Kerr et al., 2015). To determine the nitrogen-corrected apparent metabolizable energy (**AME_n_**) concentrations in turkeys, 350 day-of-hatch male Hybrid Converters poults were obtained from a commercial hatchery and placed in a single floor pen and fed a common corn-soybean meal-based diet for 8 d. On d 8, poults were placed into 56 battery cages (99 × 37 cm; 6 poults/pen) such that BW distribution was similar among cages. Dietary treatments consisted of a corn‐soybean meal basal diet containing 15% dextrose, with each DDGS treatment having DDGS substituted for the 15% dextrose (Table 2). There were 8 replications per dietary treatment. Poults were fed dietary treatments for a 6-d acclimation period followed by a 48-h balance period with total feed intake and excreta output measured over the 48-h time period. For each dietary DDGS treatment, AME_n_ was calculated using 8.73 as the nitrogen (**N**) correction factor (Titus, 1956) and subtracting the contribution of AME_n_ from dextrose•H_2_O (3,640 kcal/kg) from all dextrose-containing diets by using the following equations: total AME_n_ intake, kcal = [GE intake, kcal – GE excretion, kcal] − [8.73 × (N intake, g – N excretion, g)]; basal AME_n_ intake = AME_n_ intake of control diet (85% basal + 15% dextrose•H_2_O) − 3,640 kcal of ME/kg of dextrose•H_2_O (Anderson et al., 1958); co-product AME_n_ = (total AME_n_ intake, kcal – basal AME_n_ intake, kcal)/co-product intake, g.

**Table 1:** Analyzed composition of corn-distillers dried grains with solubles (DDGS) sources, dry matter basis.^1^

	DDGS source					
Item	A	B	C	D	E	F
Dry matter, %	88.66	88.88	89.34	89.80	90.52	91.28
Particle size, μm	544	514	491	310	338	368
Gross energy, kcal/kg	5,227	5,094	5,052	4,981	4,918	5,155
Crude protein, %	29.65	32.00	31.59	30.58	32.21	29.83
Amino acid, %						
Ala	2.00	2.21	2.08	2.20	2.24	2.05
Arg	1.34	1.50	1.35	1.46	1.38	1.29
Asp	1.83	1.98	1.87	2.02	1.99	1.91
Cys	0.52	0.56	0.54	0.63	0.59	0.58
Glu	3.79	4.17	4.06	4.64	4.60	4.31
His	0.74	0.83	0.78	0.84	0.84	0.81
Ile	1.11	1.24	1.16	1.22	1.25	1.16
Leu	3.32	3.75	3.51	3.46	3.68	3.33
Lys	1.00	1.09	1.04	1.18	1.10	1.12
Met	0.59	0.61	0.56	0.68	0.57	0.60
Phe	1.41	1.59	1.48	1.48	1.59	1.28
Pro	2.24	2.42	2.25	2.53	2.61	2.46
Ser	1.27	1.40	1.30	1.28	1.30	1.24
Thr	1.06	1.16	1.07	1.10	1.12	1.07
Trp	0.26	0.26	0.25	0.25	0.23	0.24
Tyr	1.09	1.19	1.05	1.07	1.09	1.02
Val	1.56	1.70	1.61	1.68	1.68	1.59
Total starch, %	2.50	2.33	3.82	4.93	4.40	4.68
Total dietary fiber, %	31.47	31.62	31.12	32.41	32.81	32.10
Neutral detergent fiber, %	38.27	38.49	39.58	30.95	31.05	27.84
Acid detergent fiber, %	11.48	12.14	11.60	8.90	8.55	8.55
Phosphorus, %	0.83	0.87	0.92	0.90	0.94	0.85
Ether extract, %	13.34	10.41	9.11	8.01	6.99	11.38

1 Adapted from Kerr et al., 2015.

**Table 2:** Diet formulations for determination of apparent metabolizable energy of corn-dried distillers grains with solubles (**DDGS**) in turkey poults, Experiment 1.^1^

Ingredient. %	Basal	Control	DDGS
Corn	41.71	–	–
Soybean meal	49.91	–	–
Choice white grease	0.91	–	–
Dicalcium phosphate	3.42	–	–
Limestone	2.15	–	–
Sodium chloride	0.16	–	–
Sodium sesquicarbonate	0.36	–	–
Vitamin mix^2^	0.17	–	–
Trace mineral mix^3^	0.21	–	–
Choline chloride, 60%	0.18	–	–
L-lysine·HCl	0.43	–	–
DL-methionine	0.33	–	–
L-threonine	0.06	–	–
Basal diet	–	85.00	85.00
Dextrose	–	15.00	–
DDGS sample	–	–	15.00
Total	100.00	100.00	100.00

1 Diets fed from d 8 to 16 d of age with total fecal collection occurring on d 15 and 16. Initial and final BW averaged 206 and 256 g per bird on d 14 and 16, respectively. The 48 hr feed intake averaged 87 g per bird. Dextrose was assumed to contain 3,640 kcal/kg (Hill and Anderson, 1958).

2 Provided per kg of basal diet: vitamin A, 20,613 IU; vitamin D_3_, 11,243 IU; vitamin E, 169 IU; menadione, 7.5 mg; vitamin B_12_, 94 μg; biotin, 750 μg; folic acid, 9.5 mg; niacin, 187 mg; pantothenic acid, 64 mg; pyridoxine, 11.6 mg; riboflavin, 23.8 mg; thiamine, 9.6 mg.

3 Provided per kg of basal diet: Cu, 120 mg; Fe, 48 mg; I, 4,200 μg; Mn, 192 mg; Se, 0.35 mg; Zn, 192 mg.

To determine nitrogen-corrected true metabolizable energy (**TME_n_**), 112 Hybrid Converters male turkeys (7 wks of age) were acclimated for 3 d in individual cages (30.5 × 41.2 cm) with wire flooring, nipple drinkers, and to allow clearing of any bedding residuals from the ceca prior to feeding experimental diets. Turkeys were subsequently allotted to the different treatments and fed 30 g of their respective DDGS source after a 24-hr fasting period (16 individually fed turkeys per sample). Another group of 16 turkeys continued to be fasted to determine endogenous gross energy (**GE**) and N losses. Excreta was collected underneath the cages for a 48-h time period and analyzed for GE and N, with TME_n_ determined following the methods described by Parsons (1985) and Parsons et al. (1982).

Standardized AA digestibility (**SID-AA**) was assessed by using a cecectomized chicken rooster model (Sibbald, 1976, 1985; Parsons, 1985) at the University of Illinois. In brief, 24 single Comb White-Leghorn roosters (4 roosters per DDGS sample) were individually housed in cages and fasted for 24 h. After fasting, each rooster was crop intubated and fed 30 g one of the 6 DDGS samples. Following tube-feeding, each rooster was placed in an individual wire cage over an excreta collection tray, and excreta (feces + urine) were quantitatively collected for 48 h. Excreta samples were pooled by treatment and freeze dried, weighed, and ground prior to being analyzed. Standardized AA digestibility for each DDGS sample was determined following the methods described by Parsons (1985).

Diets and digesta were analyzed for GE using an adiabatic bomb calorimeter using benzoic acid as a standard (Model 1281; Parr Instruments, Moline, IL), N using thermocombustion (Leco FP-428, Leco Corporation, St. Joseph, MI), and DM using a force air oven (Method 930.15; AOAC International, 2007), either at the University of Minnesota or at the University of Illinois. Amino acids were analyzed at a commercial laboratory (Method 982.30 E [a, b, and c]; AOAC International, 2007; University of Missouri-Columbia Experiment Station Chemical Laboratory, Columbia, MO).

### Experiment 2

For Experiment 2 (**EXP 2**), HP-DDG and CFP (ANDVantage-40Y, and ANDVantage-50Y, respectively**)** were obtained from a commercial ethanol company (The Andersons, Inc., Maumee, OH; Table 3). The process of creating HP-DDG involves selective milling technology, liquefaction, fermentation, fiber separation technology, and 3 centrifugations. CFP is made through the same process, but involves an additional liquefaction and specialized centrifuge, that removes a majority of suspended solids from the thin sillage, to reach a higher concentration of protein.

**Table 3:** Composition of high protein corn co-products, dry matter basis.

Item	HP-DDG^1^	CFP^1^
Dry matter, %	90.32	91.99
Particle size, μm	570	377
Gross energy, kcal/kg	5,575	5,597
Ether extract, %	8.89	7.57
Total starch, %	6.37	6.28
Acid detergent fiber, %	21.55	21.94
Neutral detergent fiber, %	38.06	30.98
Phosphorus, %	0.39	0.50
Crude protein, %	47.3	55.5
Amino acid, %		
Ala	3.52	3.97
Arg	2.02	2.51
Asp	3.01	3.59
Cys	0.86	1.08
Glu	7.94	9.14
Gly	1.73	2.02
His	1.38	1.63
Ile	1.98	2.29
Leu	6.11	6.83
Lys	1.37	1.92
Met	1.03	1.33
Phe	2.51	2.86
Pro	3.94	4.41
Ser	2.00	2.32
Thr	1.69	2.01
Trp	0.24	0.37
Tyr	1.92	2.23
Val	2.41	2.86

1 HP-DDG: High protein-distillers grain; CFP, corn fermented protein; The Andersons, Inc., Maumee, OH.

*Energy Digestibility:* Seventy day-of-hatch male Nicholas Select poults were obtained from a commercial hatchery (Osceola, IA), placed in a single floor pen, and fed a common corn-soybean meal-based diet for 21 d. On d 22, 60 poults were randomly selected and moved to battery cages (33 × 51 cm; one bird/pen) to determine the AME of each co-product. This was accomplished by formulating a corn-soybean meal-based basal diet containing 0.50% titanium dioxide as an indigestible marker, then formulating a control diet which contained 85% of the basal diet plus 15% dextrose or the basal diet containing one of the 2 co-products source that were added to replace dextrose (Table 4), using a methodology similar to that described for broilers (Rochell et al., 2011; Fries-Craft and Bobeck, 2019). While in the battery cages, poults had ad libitum access to feed and water. Poults were fed their respective diets for a 5-d acclimation period, followed by 4-d excreta sampling period, with 20 replications per dietary treatment. Following collection, fecal samples were dried at 55^ο^C and subsequently ground through a 1-mm screen. Feed and fecal samples were analyzed for titanium dioxide based on the method of Leone (1973), where samples were ashed in an oven and then digested with sulfuric acid and hydrogen peroxide, followed by measuring absorbance using a UV spectrophotometer against a standard curve. The GE content of feeds and excreta was determined using an isoperibol bomb calorimetry (Model 6200, Parr Instruments, Moline, IL) and benzoic acid as a standard. Apparent total tract digestibility (**ATTD**) for each diet was accomplished using the indirect method with the ATTD (%) calculated as: [1 − (Ti_feed_ × GE_exrecta_)/(Ti_excreta_ × GE_feed_)] × 100. The energy digestibility of HP-DDG and CFP was calculated by subtracting the energy contribution of the basal diet from the GE of the test diet containing the specific co-product sample, using the assumption that dextrose contains 3,640 kcal/kg (Anderson et al., 1958). Apparent metabolizable energy was calculated by multiplying ATTD for each feedstuff times its GE value.

**Table 4:** Diet formulations for determination of apparent metabolizable energy, Experiment 2.^1^

Ingredient, %	Basal	Control	HP-DDG^4^	CFP^4^
Corn	55.50	–	–	–
Soybean meal	36.74	–	–	–
Soybean oil	1.87	–	–	–
Dicalcium phosphate	3.03	–	–	–
Limestone	0.69	–	–	–
Sodium chloride	0.29	–	–	–
Vitamin mineral mix^2^	0.60	–	–	–
Choline chloride, 60%	0.17	–	–	–
L-lysine·HCl	0.27	–	–	–
DL-methionine	0.25	–	–	–
L-threonine	0.07	–	–	–
Phytase^3^	0.02	–	–	–
Titanium dioxide	0.50	–	–	–
Basal diet	–	85.00	85.00	85.00
Dextrose	–	15.00	–	–
HP-DDG^4^	–	–	15.00	–
CFP^4^	–	–	–	15.00
Total	100.00	100.00	100.00	100.00

1 Treatment diets were fed from d 22 to 31 d of age. Fecal collection occurred from d 28 to 31 d of age. Initial and final BW of 557 and 1,041 g, respectively. Feed intake across the 3 diets averaged 959 g during the 10-d feeding period. Dextrose assumed to contain 3,640 kcal/kg (Hill and Anderson, 1958).

2 Provided per kg of basal diet: vitamin A, 7,935 IU; vitamin D_3_, 2,645 IU; vitamin E, 17.2 IU; menadione, 1.0 mg; vitamin B_12_, 11 μg; biotin, 40 μg; choline, 429 mg; folic acid, 1.3 mg; niacin, 39 mg; pantothenic acid, 10 mg; pyridoxine, 1.0 mg; riboflavin, 5.3 mg; thiamine, 1.3 mg; Cu, 12 mg; Fe, 135 mg; I, 822 μg; Mn, 122 mg; Se, 0.24 mg; Zn, 121 mg.

3 Ronozyme HiPhos 2700, DSM Nutritional Products Inc., Parsippany, NJ.

4 HP-DDG: High protein-distillers grain; CFP, corn fermented protein., The Andersons, Inc., Maumee, OH.

*Amino Acid Digestibility:* Following the completion of the energy determination study, the 60 poults (32 d of age) were weighed and fed either a N-free diet or one of the two co-product test diets which were formulated to be isonitrogenous for 6 d (Table 5). On d 7 of the trial, 59 poults (38 d of age) were euthanized (one poult died on d 6 of adaptation) and ileal contents were collected. Ileal samples were pooled into 4 replicates per experimental diet (due to small sample size per individual bird), dried at 55°C in a forced-air oven, and dried samples were ground. Diets and digesta were analyzed at a commercial laboratory (University of Missouri Agricultural Experimental Station Chemistry Laboratory, Columbia, MO) for DM, CP, and AA (Methods 934.01, 990.03, and 982.30 E (a, b, c), respectively; AOAC, 2007). Standardized ileal digestibility of AA was determined as described by others (Rochell et al., 2011; Fries-Craft and Bobeck, 2019).

**Table 5:** Diet formulations for determination of standardized amino acid digestibility.^1^

Ingredient, %	Nitrogen-free	HP-DDG^3^	CFP^3^
Corn starch	41.20	20.30	25.30
Glucose	41.20	20.30	25.30
Solka floc	5.00	–	–
Soybean oil	5.00	5.00	5.00
Dicalcium phosphate	2.00	2.00	2.00
Limestone	1.00	1.00	1.00
Sodium chloride	0.20	0.20	0.20
Vitamin mineral mix^2^	0.50	0.50	0.50
Choline chloride, 60%	0.20	0.20	0.20
NaHCO_3_	2.00	–	–
KCl	1.00	–	–
MgO	0.20	–	–
Titanium dioxide	0.50	0.50	0.50
HP-DDG^3^	–	50.00	–
CFP^3^	–	–	40.00
Total	100.00	100.00	100.00

1 Diets fed from d 32 to 38 d of age. Birds were euthanized on d 38 for collection of ileal contents. Initial and final BW of 1,041 and 1,016 g, respectively. Feed intake across the 3 diets averaged 451 g during the 7-d feeding period.

2 Provided per kg of basal diet: vitamin A, 6,613 IU; vitamin D_3_, 2,205 IU; vitamin E, 14.3 IU; menadione, 0.9 mg; vitamin B_12_, 9 μg; biotin, 33 μg; choline, 358 mg; folic acid, 1.1 mg; niacin, 33 mg; pantothenic acid, 9 mg; pyridoxine, 0.9 mg; riboflavin, 4.4 mg; thiamine, 1.1 mg; Cu, 10 mg; Fe, 113 mg; I, 685 μg; Mn, 102 mg; Se, 0.20 mg; Zn, 100 mg.

3 HP-DDG: High protein-distillers grain; CFP, corn fermented protein. The Andersons, Inc., Maumee, OH.

*Growth Performance Evaluation:* Diets for 2 feeding phases were formulated using the previously determined AME and SID-AA values with all diets formulated to be isoenergetic and isonitrogenous, Table 6. The feeding regimen included a control diet that was formulated to mimic standard commercial poult diets (6 pens); HP-DDG diets that contained either 7.5% inclusion of HP-DDG (6 pens) or 15% inclusion of HP-DDG (7 pens); and CFP diets that contained 7.5% inclusion of CFP (6 pens) or 15% inclusion of CFP (7 pens). Percent inclusion rates were based on previous research of DDGS in turkeys (Couch et al., 1957; Potter, 1966; Noll and Brannon, 2006).

**Table 6:** Phase 1 and Phase 2 diet formulation for the growth performance trial, Experiment 2.^1^

	Phase I^2^					Phase II^3^				
	Control	HP-DDG^4^		CFP^4^		Control	HP-DDG^4^		CFP^4^	
Item, %	0	7.5	15	7.5	15	0	7.5	15	7.5	15
Corn	37.35	35.59	33.51	38.60	39.51	39.21	38.35	36.33	41.46	42.34
Soybean meal	45.95	39.99	34.31	38.14	30.61	45.10	38.29	32.53	36.35	28.84
Poultry meal	7.50	7.50	7.50	7.50	7.50	7.50	7.50	7.50	7.50	7.50
Soybean oil	4.23	4.32	4.45	3.17	2.15	3.75	3.72	3.85	2.55	1.55
HP-DDG^4^	-	7.50	15.00	-	-	-	7.50	15.00	-	-
CFP^4^	-	-	-	7.50	15.00	-	-	-	7.50	15.00
Limestone	0.72	0.82	0.92	0.83	0.94	0.70	0.80	0.90	0.81	0.92
Dicalcium phosphate	2.43	2.31	2.19	2.32	2.22	1.96	1.85	1.73	1.86	1.75
Vitamin-mineral mix^5^	0.30	0.30	0.30	0.30	0.30	0.30	0.30	0.30	0.30	0.30
Choline chloride, 60%	0.30	0.30	0.30	0.30	0.30	0.30	0.30	0.30	0.30	0.30
NaCl	0.29	0.29	0.29	0.29	0.29	0.29	0.29	0.29	0.29	0.29
DL-methionine	0.42	0.39	0.35	0.36	0.29	0.41	0.39	0.35	0.36	0.29
L-lysine·HCl	0.36	0.48	0.59	0.48	0.59	0.33	0.48	0.59	0.48	0.59
L-threonine	0.12	0.13	0.13	0.12	0.12	0.12	0.14	0.15	0.13	0.13
L-arginine	-	0.05	0.12	0.06	0.14	-	0.07	0.15	0.08	0.16
Phytase^6^	0.04	0.04	0.04	0.04	0.04	0.04	0.04	0.04	0.04	0.04
TOTAL	100.00	100.00	100.00	100.00	100.00	100.00	100.00	100.00	100.00	100.00
Calculated Analysis										
Crude Protein	28.16	32.72	29.63	29.37	29.77	27.86	30.06	28.78	28.63	29.28
Moisture	10.75	9.69	9.96	9.79	10.06	10.50	10.96	10.59	11.04	10.82
Crude Fat	3.66	3.91	5.78	3.89	3.95	4.25	4.72	4.97	3.57	3.53
Crude Fiber	2.38	3.04	3.32	2.67	2.65	3.04	3.11	3.54	2.73	2.99
Ash	8.08	8.33	7.89	8.23	7.09	7.39	6.96	6.62	7.43	6.50

1 Phase 1 diets fed from d 1 to 14 d of age and Phase 2 diets fed from d 15 to 32 day of age. Average d 1, 14, and 32 BW were 64, 397, and 1,665 g, respectively.

2 Phase I diets were formulated to 2,950 kcal AME/kg, 1.35% calcium, 0.64% nonphytate phosphorus, and 1.75 ileal digestible Lys with minimum ileal digestible TSAA, Thr, Trp, Ile, Val, and Arg to Lys ratios of 0.650, 0.580, 0.140, 0.600, 0.660, and 1.020, respectively.

3 Phase II diets were formulated to 2,950 kcal AME/kg, 1.24% calcium, 0.57% nonphytate phosphorus, and 1.71 ileal digestible Lys with minimum ileal digestible TSAA, Thr, Trp, Ile, Val, and Arg to Lys ratios of 0.660, 0.590, 0.150, 0.600, 0.670, and 1.030, respectively.

4 High protein-distillers grain (HP-DDG) or corn fermented protein (CFP), The Andersons, Inc., Maumee, OH.

5 Provided per kg of basal diet: vitamin A, 16,534 IU; vitamin D_3_, 5,556 IU; vitamin E, 33 IU; menadione, 5.6 mg; vitamin B_12_, 19 μg; biotin, 73 μg; folic acid, 0.7 mg; niacin, 102 mg; pantothenic acid, 18 mg; pyridoxine, 4.0 mg; riboflavin, 11.2 mg; thiamine, 1.3 mg; Cu, 14 mg; Fe, 138 mg; I, 1,500 μg; Mn, 105 mg; Se, 0.30 mg; Zn, 105 mg.

6 Ronozyme HiPhos 2700, DSM Nutritional Products Inc., Parsippany, NJ.

On the day of hatch, 1,800 male Nicholas Select poults from a commercial hatchery were weighed and randomly placed across 32 floor pens in the Stanley Balloun Turkey Teaching and Research Barn, Ames, IA. Upon placement, poults received their assigned experimental diets along with electrolytes in the drinking water (Balance, Aurora Pharmaceutical, Inc., Northfield, MN) for 3 d. Birds were managed under commercial poult brooder conditions and fed a 2-phase brooder diet that was switched on d 14. Poults and feed were weighed at placement, d 14 and d 32. All feed added to the pens was weighed, and daily mortality was counted and weighed by pen. Mortality adjusted feed conversion ratio (**FCR**) was calculated for phase I (0-14 d), phase II (15–32 d) and throughout the brooder phase (0–32 d) using the formula: (feed added - feed disappearance)/(starting weight – [ending weight + mortality weight]).

*Poult Intestinal Permeability:* Intestinal permeability was measured using methods described by Baxter et al. (2017). On d 31, 2 poults per pen were randomly chosen for poult intestinal health measurements. Two pens (1 pen each from 15% HP-DDG and 15% CFP diets) were used to collect blood samples used as control serum. In these pens, poults were euthanized without oral gavage of fluorescein isothiocyanate dextran (**FITC-D,** Sigma Aldrich, St. Louis, MO, FD4). All other selected poults were orally gavaged with 8.32 mg FITC-D/kg BW dissolved in distilled water. After FITC-D administration, poults were marked with animal safe paint to denote treatment group and were returned to their home pen. One hour after gavage, poults were euthanized via carbon dioxide asphyxiation. Poults were individually weighed, and blood samples were collected from the femoral artery and placed into serum blood collection tubes (BD Medical, Franklin Lakes, NJ). Blood was allowed to clot at room temperature, then centrifuged at 1,000 × *g* for 15 min. Following separation, serum was allocated into 0.5 mL aliquots and stored in amber tubes at -80°C until analysis.

Serum from poults that did not receive the FITC-D treatment were combined to create a control serum solution, diluted at a 1:5 ratio with PBS and used to generate a standard curve. Thawed serum was diluted at a 1:5 ratio using PBS and plated in triplicate on a black 96-well plate. Fluorescence was measured using a spectrophotometer (BioTek Cytation, BioTek US, Winooski, VT) with excitation and emission wavelengths of 485 and 528 nm, respectively. Data were averaged per poult for statistical analysis.

*Poult Intestinal Morphology:* Following blood collection, a 1 cm section of the duodenum at the duodenum loop, jejunum at Meckel's Diverticulum, and ileum 2 cm proximal to the ileocecal junction were excised. Intestinal segments were preserved in 10% buffered formalin solution before being transported to the Veterinary Histopathology Laboratory at Iowa State University (Ames, IA) where samples were sectioned, embedded in paraffin, fixed onto slides, and stained with hematoxylin and eosin stains. Villus height and crypt depth measurements were recorded using an Olympus BX63 microscope and camera (Olympus Corporation, Tokyo Japan). Five to 10 morphometric measurements were recorded per sample based on section quality. Data were averaged for each poult by parameter for statistical analysis.

### Statistical Analysis

*Experiment 1:* The pen and individual bird were considered as the experimental unit for the AME_n_ and TME_n_ data, respectively. The AME_n_ and TME_n_ data were analyzed using PROC MIXED (SAS Institute Inc., 2013) as a completely randomized design with pen considered as a random variable. Because the excreta for the 4 individually fed roosters was pooled into a single sample prior to analysis, data for SID-AA are reported as mean values only.

*Experiment 2:* The data for AME were analyzed using the pen (1 bird/pen) as the experimental unit within the completely randomized experimental design using PROC Glimmix (SAS Institute Inc., 2013). Because excreta were pooled from 2 to 5 birds per treatment to obtain enough samples for AA analysis, data for SID-AA are reported as mean values only. Differences for poult growth performance, intestinal permeability, and intestinal morphology were determined between treatment groups using PROC Glimmix in SAS (SAS Institute Inc., 2013) with dietary type (Control, HP-DDG, or CFP), diet inclusion rate (0, 7.5, or 15%), and the interaction for dietary type and inclusion rate were fit as a fixed. Following analysis, residuals were tested for normality and homoscedasticity using Proc univariate (SAS Institute Inc., 2013). Significance was set at *P* < 0.05 and if significance criteria was met, pairwise comparisons were determined using the pdiff statement in SAS (SAS Institute Inc., 2013).

## RESULTS

### Experiment 1

Values for AME_n_, TME_n_, and the standardized ileal digestibility of AA for the 6 DDGS samples used in EXP 1 are shown in Table 7. In turkey poults, the AME_n_ among the 6 DDGS ranged from 2,530 to 3,573 kcal/kg DM and was not found to be different (*P* = 0.57) among the samples evaluated, largely due to the SEM of 476 observed in this trial. In contrast, TME_n_ ranged from 2,747 to 3,138 kcal/kg DM with differences observed among the DDGS samples evaluated (*P* = 0.01). Diet F having the larger TME_n_ compared to B, C, and D. Using cecectomized roosters, the lowest SID-AA were observed for LYS (66.62%), THR (74.09%), and ASP (74.56%) while the highest SID-AA were observed for LEU (89.41%), PRO (87.45%), and TRP (87.17%), with other SID-AA being intermediate (Table 7).

**Table 7:** Apparent metabolizable energy corrected for nitrogen retention (AME_n_) and true metabolizable energy corrected for nitrogen retention (TME_n_) determinations in young turkeys and standardized amino acid digestibility of distillers dried grains with solubles (DDGS) in cecetomized roosters, dry matter basis in experiment 1.

Sample	A	B	C	D	E	F	SEM	P value
AME_n_, kcal/kg^1^	3,346	2,530	2,767	2,606	3,573	2,853	476	0.57
TME_n_, kcal/kg^2^	2,947^ab^	2,747^b^	2,784^b^	2,761^b^	2,811^ab^	3,138^a^	79	0.01
Amino acid^3^, %								
Ala	86.49	84.61	84.90	84.56	83.57	87.22	NA	NA
Arg	88.25	84.48	85.46	85.60	82.77	88.01	NA	NA
Asp	77.39	73.44	72.98	73.95	71.14	78.43	NA	NA
Cys	78.20	74.93	75.46	83.47	73.93	84.14	NA	NA
Glu	87.37	85.17	86.37	85.99	84.74	88.41	NA	NA
His	83.41	82.85	82.73	81.26	80.89	84.73	NA	NA
Ile	84.64	81.53	82.03	81.30	80.12	83.80	NA	NA
Leu	90.98	88.91	89.63	88.19	88.28	90.48	NA	NA
Lys	68.48	65.23	64.00	66.51	62.32	73.19	NA	NA
Met	87.87	85.41	84.98	88.14	84.29	88.83	NA	NA
Phe	86.78	84.03	84.25	83.90	83.77	85.18	NA	NA
Pro	88.68	86.25	88.01	86.82	85.49	89.46	NA	NA
Ser	83.39	80.40	80.21	78.60	76.76	85.05	NA	NA
Thr	77.73	74.89	73.18	71.48	69.24	78.00	NA	NA
Trp	90.43	86.94	87.29	86.29	81.67	90.42	NA	NA
Tyr	88.05	84.81	84.45	82.99	82.37	86.56	NA	NA
Val	85.35	81.65	83.04	81.91	79.26	83.40	NA	NA

1 AME_n_ represents the mean of 8 pens of 6 poults/pen per DDGS sample.

2 TME_n_ represents the mean of 16 individually fed turkeys per DDGS sample.

3 The standardized amino acid digestibility data represent 4 individually fed roosters with excreta pooled to a single sample prior to analysis such that no statistical analysis is reported.

ab Superscripts represent significant differences (P < 0.05). Abbreviations: NA, not applicable; SEM, standard error of the mean; P, model probability value.

### Experiment 2

*Energy and Amino Acid Digestibility:* Values for AME and SID-AA for the HP-DDG and CFP samples are shown in Table 8. The AME of CFP was greater than HP-DDG (*P* = 0.001), with its SID-AA being approximately 6% greater in the CFP sample compared with those in the HP-DDG sample. The SID were comparable for LYS (66.55 and 77.00%), MET (85.99 and 89.78%), and THR (71.92 and 77.78%) for HP-DDG and CFP, respectively.

**Table 8:** Apparent metabolizable energy determinations in young turkeys and standardized amino acid digestibility (**SID-AA**) of high protein corn co-products in young poults, dry matter basis in Experiment 2.

DDG sample	HP-DDG^1^	CFP^1^	SEM	P value
AME, kcal/kg^2^	3,313	4,000	95	0.001
AME_n_, kcal/kg^3^	3,114	3,760	NA	NA
Amino acid	SID-AA, %^4^	SID-AA, %^4^	E-AA, mg/kg^5^	
Ala	85.82	88.17	233	
Arg	81.63	86.14	193	
Asp	72.79	77.13	416	
Cys	74.19	80.16	189	
Glu	86.70	89.51	498	
Gly	70.92	78.14	265	
His	79.96	85.18	124	
Ile	80.09	83.67	208	
Leu	88.61	90.69	317	
Lys	66.55	77.00	208	
Met	85.99	89.78	65	
Phe	85.18	88.07	214	
Pro	85.12	88.49	324	
Ser	78.77	82.80	300	
Thr	71.92	77.78	367	
Trp	67.78	78.82	65	
Tyr	84.33	87.65	176	
Val	79.57	83.60	265	

1 HP-DDG: High protein-distillers grain; CFP, corn fermented protein, The Andersons, Inc., Maumee, OH.

2 Apparent metabolizable energy (AME) based on 20 individually fed poults per treatment. Poults fed the basal diet including glucose exhibited an AME of 2,999 kcal/kg as-is basis. Both test ingredients were included at 15%.

3 AME_n_ was calculated by reducing AME by 6% based on Applegate et al., 2009; Cozannet et al., 2010; Adeola and Zhai, 2012; Ning et al., 2014; Adebiyi and Olukosi, 2015; Dalolio et al., 2020; Abdollahi et al., 2021; Dias et al., 2023. Statistics not applicable (NA) because values calculated from AME values.

4 The standardized ileal amino acid digestibility (SID-AA) data represent 4 samples per co-product source with excreta pooled (2 to 5 birds/sample) to acquire enough sample for AA analysis such that no statistical analysis is reported.

5 Endogenous amino acids (E-AA) based on 3 pooled samples from poults fed the nitrogen free diet. Abbreviations: NA, not applicable; SEM, standard error of the mean; P, model probability value.

*Poult Growth Performance and Mortality:* Growth performance and mortality data of poults fed diets containing no co-products or diets with 7.5% or 15% HP-DDG or CFP are presented in Table 9. Body weights were similar between poults fed diets containing the different co-product sources, inclusion rates, and their interaction at placement, 14 d, and 32 d (*P* > 0.10). Mortality number was similar between poults fed diets containing the 2 high protein co-product samples, their inclusion rate, and their interaction during the first 14 d (*P* > 0.70). In contrast, differences in poult mortality number were observed due to feeding poults diets with different co-product inclusion rates between 14 d and 32 d (*P* < 0.01) with birds fed diets containing 15% HP-DDG or CFP having a higher mortality rate compared to birds fed diets containing 0 and 7.5% high protein corn co-products. Mortality number were similar between poults fed diets containing the co-product sources, and there was no interaction between the diet inclusion rates and co-product source between 14 d and 32 d (*P* = 0.28 and *P* = 0.28; respectively). Mortality over the entire trial period was not impacted by co-product source, diet inclusion rate, or the interaction between co-product source and diet inclusion rate (*P* > 0.24).

**Table 9:** Growth performance of poults fed increasing diet inclusion rates of high protein corn co-products^1^.

	Control	HP-DDG^2^, %		CFP^2^, %		Statistics^3^			
Criterion	0	7.5	15	7.5	15	SEM	Source	Inclusion rate	Interaction
Phase I, d 1-14									
Body weight, kg	0.40	0.39	0.40	0.40	0.40	*0.01*	0.17	0.57	0.26
Feed intake, kg	0.46^a^	0.41^b^	0.45^a^	0.46^a^	0.45^a^	*0.01*	0.02	0.12	0.04
FCR	1.37	1.25	1.35	1.36	1.35	*0.03*	0.04	0.08	0.06
Mortality	0.83	0.83	0.71	0.67	0.88	*0.45*	0.98	0.94	0.73
Phase II, d 15-32									
Body weight, kg	1.69	1.66	1.66	1.67	1.64	*0.02*	0.63	0.22	0.32
Feed intake, kg	1.80	1.91	1.97	1.95	1.91	*0.05*	0.83	0.87	0.31
FCR	1.40	1.51	1.58	1.56	1.55	*0.04*	0.83	0.43	0.38
Mortality	0.00	0.00	0.43	0.00	0.86	*0.20*	0.28	0.01	0.28
Overall									
Body weight, kg	1.69	1.66	1.66	1.67	1.67	*0.02*	0.63	0.22	0.32
Feed intake, kg	2.26	2.32	2.42	2.41	2.36	*0.06*	0.64	0.19	0.77
FCR	1.40	1.48	1.53	1.51	1.53	*0.04*	0.35	0.60	0.63
Mortality	0.83	0.83	1.14	0.67	1.71	*0.57*	0.24	0.52	0.72

1 Phase 1 diets fed from d 1 to 14 d of age and Phase 2 diets fed from d 15 to 32 day of age. Average d 1, 14, and 32 body weights were 64, 397, and 1,665 g, respectively.

2 High protein-distillers grain (HP-DDG) or corn fermented protein (CFP), The Andersons, Inc., Maumee, OH.

3 There were 56 birds per pen with 6 replications for birds fed the control, 7.5% HP-DDG, and 7.5% CFP dietary treatments and 7 replications for birds fed the 15% HP-DDG and 15% CFP dietary treatments.

ab Superscripts represent significant differences (*P* = 0.05).

Feed intake was altered by co-product source, resulting in an interaction between the co-product diet inclusion rate and source during the first 14 d (*P* = 0.02 and *P* = 0.04, respectively; Table 9). Feed intake was less in poults fed diets containing 7.5% HP-DDG compared to poults fed all other diets. However, between 14 d and 32 d, poult feed intake was similar regardless of co-product source and diet inclusion rate (*P* > 0.30). Feed intake over the entire trial period was not impacted by co-product source and diet inclusion rate, and there was no interaction between co-product source and inclusion rate (*P* > 0.24).

Feed conversion ratio was significantly altered by dietary co-product source (*P* = 0.04) with diets containing HP-DDG having a more favorable FCR compared to poults fed either no co-product or diets containing CFP during the first 14 d. In addition, during the first 14 d, dietary inclusion rate of the co-product and the interaction between co-product inclusion rate and co-product source tended to be different (*P* = 0.08 and *P* = 0.06; respectively). However, from 14 d to 32 d, and for the entire trial period, differences were not observed in FCR between co-product sources, diet inclusion rates, and nor were there interactions between source and inclusion rate (*P* > 0.10; Table 9).

*Poult Intestinal Health Measurements:* Co-product source and diet inclusion rate no effect on intestinal permeability, villi height, crypt depth, or their ratio in the jejunum and duodenum (*P* > 0.05; Table 10). Ileal villus height and the ratio of the villus height to crypt depth were altered by the dietary co-product source for villus height and the villi height:crypt depth ratio (*P* = 0.01, Table 10), with poults fed diets containing CFP having a lower villus height and villi height:crypt depth ratio compared to poults fed the control and HP-DDG dietary treatments. Ileal crypt depth was not altered by dietary co-product source, inclusion rate or the interaction between source and inclusion rate (*P* > 0.05; Table 10).

**Table 10:** Intestinal morphology and permeability of poults fed high protein corn co-products^1^.

	Control	HP-DDG^2^, %		CFP^2^, %		Statistics			
Criterion	0	7.5	15	7.5	15	SEM	Source	Inclusion rate	Interaction
Duodenum									
Villi height, µm	1,881	1,842	1,981	1,907	2,009	*159.7*	0.78	0.46	0.91
Crypt depth, µm	81.3	71.6	64.1	70.0	67.8	*7.46*	0.89	0.52	0.72
Villi:crypt ratio	26.80	26.15	29.99	28.34	31.67	*3.17*	0.55	0.27	0.94
Jejunum									
Villi height, µm	815	876	872	857	812	*83.4*	0.64	0.77	0.81
Crypt depth, µm	62.0	61.5	54.7	64.3	60.2	*4.78*	0.39	0.25	0.78
Villi:crypt ratio	13.73	15.16	15.85	14.63	13.60	*1.62*	0.40	0.92	0.60
Ileum									
Villi height, µm	637	583	607	460	513	*41.2*	0.01	0.35	0.71
Crypt depth, µm	69.7	57.0	60.8	58.4	59.7	*4.84*	0.97	0.60	0.80
Villi:crypt ratio	9.53	10.92	10.26	8.23	8.68	*0.85*	0.01	0.90	0.51
Permeability									
FITC-D^3^, ng/mL	737	890	874	834	906	*60.7*	0.84	0.65	0.47

1 Phase 1 diets fed from d 1 to 14 d of age and Phase 2 diets fed from d 15 to 32 day of age. Samples obtained from 2 birds/pen on d 31. There were with 6 replications for birds fed the control, 40Y-7.5%, and 50Y-7.5% dietary treatments and 7 replications for birds fed the 40Y-15% and 50Y-15% dietary treatments.

2 High protein-distillers grain (HP-DDG) or corn fermented protein (CFP), The Andersons, Inc., Maumee, OH.

3 Fluorescein isothiocyanate dextran.

## DISCUSSION

Two-thirds of the cost to raise a turkey comes from feed ingredients (US Poultry Industry Manual, 2022). The inclusion of alternative feed ingredients, such as DDGS or DDG, has been shown to maintain growth performance while decreasing the overall cost of poultry production (Dinani et al., 2018; Jang et al., 2022). While there are AME_n_ and SID-AA data on DDGS, HP-DDG, and CFP in broilers or roosters (Corray et al., 2019; Fries-Craft and Bobeck, 2019; Burton et al., 2021), data are limited on feeding these co-products to turkeys (Roberson, 2003; Dinani et al., 2018; Burton et al., 2021; Jang et al., 2022). As a result, this study explored the use of these corn co-products as an energy and AA source in turkey poult diets.

### Metabolizable Energy

The lack of significant differences in AME_n_ concentrations among the 6 DDGS samples in EXP. 1 was not unexpected given the experimental SEM of 476 (Table 7), which could be due to sampling, analytical, and biological errors, thereby masking potential energy differences. Because the DDGS samples used in EXP. 1 were the same samples used in a previous broiler study (Meloche et al., 2014) and finishing pig study (Kerr et al., 2015), a comparison of these values is of interest. On average, the 6 DDGS samples had a higher AME_n_ in turkey poults (16 d of age; 2,946 kcal/kg DM) compared with broilers (18 d of age; 2,816 kcal/kg DM), but the direction and magnitude of these differences among the samples were inconsistent between poults and broilers (Table 11). Although there are no recent comprehensive literature reviews on AME_n_ concentrations in corn co-products fed to poultry, Rochell (2018) reviewed the broiler AME_n_ data conducted on DDGS by the University of Auburn (Rochell et al., 2011; Meloche et al., 2013; 2014) and Jie et al. (2013) summarized rooster TME data from 28 sources of DDGS in China. Both researchers concluded that ether extract, dietary fiber, and ash concentration of DDGS are essential components of an energy prediction model, similar to that reported in swine (Anderson et al., 2012; Kerr et al., 2013; 2015). While it has been stated that ether extract might be a central component of predicting the AME (poultry) or ME (swine), this is clearly not the case because fiber concentrations have a greater impact than lipid concentration and digestibility in determining AME and ME concentrations among DDGS sources with variable composition (Rochell, 2018; Urriola et al., 2014). The same DDGS samples fed to finishing pigs had greater ME values (average of 3,795 kcal/kg DM) compared with those in either turkeys or broilers among the 6 DDGS samples evaluated in the current study. This was expected because pigs have a greater lower gut fermentation capacity to produce short chain fatty acids that contribute energy, compared with poults or broilers (Jørgensen et al., 2007; Karasov and Douglas, 2013; Lilburn and Loeffler, 2015; Ravindran and Abdollahi, 2021).

**Table 11:** Apparent metabolizable energy comparisons between poults, broilers, and pigs fed the same dried distillers dried grain samples, dry matter basis.

Sample	A	B	C	D	E	F
AME_n_, kcal/kg^1^	3,346	2,530	2,767	2,606	3,573	2,853
AME_n_, kcal/kg^2^	3,634	2,553	2,869	2,781	2,523	2,535
ME, kcal/kg^3^	3,830	3,723	3,874	3,716	3,734	3,893

1 AME_n_ = apparent metabolizable energy corrected for nitrogen of male poult (14-16 d of age) in Experiment 1.

2 AME_n_ of male broilers (16-18 d of age) from Meloche et al. (2014).

3 ME = metabolizable energy of female pigs (93 kg body weight) from Kerr et al. (2015).

In EXP 2, both HP-DDG and CFP had greater AME_n_ (adjusted values of 3,114 and 3,760 kcal/kg DM; respectively) concentrations compared to the average of the 6 DDGS samples evaluated in EXP 1 (2,946 kcal/kg DM). This is supported by results reported by Rochell et al. (2011) showing that AME_n_ concentration in HP-DDG was greater than in conventional DDGS, and data reported by Parsons et al. (2023) showing that a reduced fiber HP-DDGS had a greater TME concentration compared with that in conventional DDGS. However, Adeola and Zhai (2012) reported that conventional DDGS had greater AME_n_ content compared with that in DDG, which the authors suggested may have been due to the amount of solubles added back to the coarse grains fraction during the production process, and are known to have substantial lipid content (Kim et al., 2008; Anderson et al., 2012).

A brief summary of data evaluating corn co-products used in turkey diets is presented in Table 12. Except for the 6 DDGS samples evaluated in EXP 1 and the 10 wheat-DDGS samples evaluated by Cozannet et al. (2010), all other data that have been reported in published studies were based on evaluating only a single co-product sample. Because of the scarcity of such data, broiler or laying hen data are often used for predicting the energy value of feedstuffs used in the formulation of turkey diets. It is worthy to note that Dale and Fuller (1980) reported that TME values were approximately 3% lower in broilers compared to roosters or poults when comparing commonly used feedstuffs or complete diets. In contrast, Cozannet et al. (2010) reported that AME_n_ values were approximately 6% lower in turkeys compared with those of broilers or roosters when comparing 10 sources of wheat DDGS. This is supported by Adebiyi and Olukosi (2015) who reported that AME_n_ values were approximately 6% lower in turkeys compared to broilers when comparing values for a single wheat DDGS source. While not peer-reviewed, Banks (2011) reported that AME_n_ values averaged 4% less in turkeys compared with broilers when comparing 2 high protein distillers grains sources, but differed widely depending on if it was HP-DDG or HP-DDGS, with both turkey and broiler values being substantially less than the AME values determined in laying hens.

**Table 12:** Abbreviated summary of the composition and the energy and amino acid digestibility of turkeys fed corn- or wheat-based co-products, dry matter basis.

	Exp 1^1^	Exp 2^2^		Banks^3^		Abebiya^4^	Cozannet^5^
Sample	DDGS	HP-DDG	CFP	HP-DDGS	HP-DDG	wDDGS	wDDGS
Composition, %							
Dry matter	89.7	90.3	92.0	94.9	96.9	85.8	92.6
Crude protein	31.0	47.3	55.5	48.1	57.8	38.0	36.1
Arg	1.39	2.02	2.51	1.69	1.77	1.38	-
Ile	1.19	1.98	2.29	2.00	2.42	1.60	-
Lys	1.09	1.37	1.92	1.03	0.98	0.90	-
Met	0.60	1.03	1.33	1.03	1.34	0.52	-
Thr	1.10	1.69	2.01	1.72	1.97	1.34	-
Trp	0.25	0.24	0.37	0.32	0.35	0.44	-
Ether extract	9.9	8.9	7.6	2.7	1.9	8.7	4.6
Neutral detergent fiber	34.4	38.1	31.0	27.9	41.7	45.3	29.2
Acid detergent fiber	10.2	21.6	21.9	16.0	26.3	26.0	12.0
Total starch	3.8	6.4	6.3	-	-	-	4.1
Digestibility							
AME, kcal/kg	3,134^6^	3,313	4,000	3,596	2,153	3,355	2,316
AME_n_, kcal/kg	2,946	3,114^6^	3,760^6^	2,968	1,656	3,109	2,165
SID AA, %							
Arg	85.8	81.6	86.1	80.4	75.2	ND	ND
Ile	82.2	80.1	83.7	79.3	72.5	ND	ND
Lys	66.6	66.6	77.0	63.5	52.5	ND	ND
Met	86.6	86.0	89.8	86.4	82.5	ND	ND
Thr	74.1	71.9	77.8	69.5	60.6	ND	ND
Trp	87.2	67.8	78.8	79.1	70.6	ND	ND

1 Experiment 1: apparent metabolizable energy (AME) or AME corrected for nitrogen (AME_n_) data represents an average of 6 corn-distillers dried grains with solubles (DDGS) samples fed to young male poults, 14-16 d of age while the data for standardized ileal digestibility of amino acids (SID-AA) is based on rooster assay.

2 Experiment 2: data represent high protein-distillers grain (HP-DDG) or corn fermented protein (CFP), The Andersons, Inc., Maumee, OH.) fed to young male poults, 28-31 d of age.

3 Banks 2011: data represents 1 corn-based HP-DDGS or HP-DDG fed to young male poults, 20-21 d of age.

4 Abebiya and Olukosi, 2015: data represent one wheat-DDGS sample fed to young male poults, 14-21 d of age.

5 Cozannet et al., 2010: data represent an average of 10 wheat-DDGS samples fed to male turkeys, 86-88 d of age.

6 Data represent a 6% adjustment based on Applegate et al., 2009; Cozannet et al., 2010; Adeola and Zhai, 2012; Ning et al., 2014; Adebiyi and Olukosi, 2015; Dalolio et al., 2020; Abdollahi et al., 2021; Dias et al., 2023.

Even though Rochell (2018) conducted a partial review of the caloric value of corn co-products in broilers, although a more detailed summary and meta-analysis of corn co-product data in all types of poultry is needed, similar to that conducted in swine (Urriola et al., 2014). In our current study, we reported both AME and AME_n_ values with an adjustment factor of 6% based on corn- or wheat-based co products (Applegate et al., 2009; Cozannet et al., 2010; Adeola and Zhai, 2012; Ning et al., 2014; Adebiyi and Olukosi, 2015; Dalólio et al., 2020; Abdollahi et al., 2021; Dias et al., 2023). The TME values were also determined for the 6 DDGS samples used in EXP 1. Unlike the AME_n_ values, however, differences in TME_n_ values for these samples were observed, but the direction and the magnitude of the differences among the samples compared to AME_n_ were inconsistent. While AME, AME_n_, and TME_n_ values are provided, a detailed discussion of AME versus AME_n_ or AME versus TME (Muztar and Slinger, 1981a,b; Wolynetz and Sibbald, 1984; Sibbald, 1985) and a discussion of energy systems for poultry (Farrell, 1999; Mateos et al., 2019; Wu et al., 2020; Abdollahi et al.,2021; Noblet et al., 2022) is provided elsewhere.

### AA Digestibility

The fact that differences exist in AA digestibility values, and caloric values, within a specific classification of feedstuffs (i.e., DDGS-Table 7) and between feedstuffs (i.e., HP-DDG and CFP-Table 8) should be common knowledge. The data provided herein are useful for updating composition and digestibility databases such as the National Animal Nutrition Program database (https://animalnutrition.org/) and the NRC (1994). Regardless, some comparisons are deemed noteworthy whereupon Table 12 provides an abbreviated summary of AA digestibility from feeding DDGS (EXP 1), HP-GGE and CFP (EXP 2), or HP-DDGS and HP-DDG (Banks, 2011) to turkeys. In the current experiment, SID-AA digestibility in HP-DDG were less than in CFP supporting the notion that CFP not only had a greater concentration of AA (Table 3), but they were more digestible (Table 8). Comparatively speaking, the DDGS samples evaluated in EXP 1 generally had an average AA digestibility intermediate between HP-DDG and CFP. Compared to SID-AA digestibility for HP-DDGS and HP-DDG reported by (Banks, 2011), the SID-AA digestibility values for DDGS (EXP 1-rooster assay) and for HP-DDG and CFP (EXP 2-poult assay) samples used in the current studies were both higher compared to those reported by (Banks, 2011). The current experiment (EXP 2) is in contrast to Banks (2011) who reported that not only do HP-DDGS had greater AA concentrations, but they also have greater SID-AA digestibility compared to HP-DDG, a contrast for which cannot be explained. Similar to extrapolation of energy values of ingredients derived from broilers studies for turkeys, broiler or rooster data are also heavily relied upon for estimating SID-AA digestibility of ingredients when formulating diets for turkeys. Although the ranking of digestibility of individual AA are often similar between turkeys and roosters, differences are not always in a consistent direction or magnitude depending on feedstuff evaluated (Firman and Remus, 1993; Kluth and Rodehutscord, 2006; Adedokun et al., 2008). However, AA digestibility data derived from the rooster assay and ileal digestibility assays are similar and may likely be interchangeable among species of birds (Tahir and Pesti, 2012; Parsons, 2020). Based on the interchangeable of AA digestibility among species of birds and on a meta-analysis of the data reported by Zhu et al. (2018), the total AA content appears to the best predictor of SID AA digestibility of AA for poultry based on regression coefficients.

### Performance and Intestinal Function

While Mustafa et al. (2017) observed no effects from feeding diets containing HP-DDG on broiler growth performance, high inclusion rates of DDGS or HP-DDG have been observed to decrease turkey (18%, Roberson, 2003) and broiler (18%, Lumpkins et al., 2004; 25%, Wang et al., 2007), growth performance. Some researchers have suggested that one of the potential reasons for this reduced growth performance in swine and poultry when fed diets with high amounts of DDGS may be due to the increased NSP concentrations that may disrupt intestinal health. No data have been published regarding the impact of feeding diets containing HP-DDG or CFP on turkey growth performance and intestinal health. In the current study, inclusion of 7.5 or 15% HP-DDG or CFP in diets fed to young turkeys (32 d of age) did not affect overall poult growth rate, feed intake, feed conversion, or mortality. These results are similar to those reported by Burton et al. (2021) which showed that feeding turkey poults diets containing 4 or 8% CFP had no impact on performance compared to poults fed control diets with no corn co-products.

While there were no differences in growth performance during the entire 32 d feeding period, small differences were observed for feed intake and FCR during the first 14 d post placement. During the first 14 d of feeding, feed intake was reduced in poults fed diets containing HP-DDG, which mainly occurred in birds fed the 7.5% HP-DDG diet, but there was no significant source by diet inclusion rate interaction. However, by the time the study was concluded at 32 d, feed intake was similar among all dietary treatments. Based on work by Krogdahl and Sell (1989) pancreatic zymogen production of proteases, lipases, and amylases are low during the first 16 to 24 d of life, which is also closely associated with the time in which birds start increasing their average daily gain. Therefore, this short-term reduction in feed intake during this initial feeding period may have been due to delayed or less production of these pancreatic zymogens in birds fed the HP-DDG diets. The return to control levels for feed intake and FCR observed in this study would suggest the decrease in feed intake and improvement in FCR is temporary.

During the second portion of the study (14–32 d), mortality in pens fed diets containing 15% HP-DDG or CFP had higher mortality compared to poults fed the other dietary treatments. That said, this study as a whole had very limited morality and this significant difference was limited to very low numbers of poults. However, mortality across the whole brooder period were not significantly different. The results reported herein are supported by Farahat et al. (2013) who reported a higher mortality from weeks 2 through 5 (2.93% compared to 0.28%) in poult fed a 20% inclusion rate of DDGS, an observation which needs to be clarified in future research. Because AA digestibility and growth performance were similar among birds fed the HP-DDG and CFP diets, no changes in intestinal morphology and permeability would be expected. Therefore, we observed no effects of co-product source or diet inclusion rate on intestinal permeability as measured by the macromolecule flux of FITC-D, and intestinal morphology of the major sections of the intestine responsible for digestion (duodenum and jejunum) and absorption (jejunum). However, in the ileum, the villus height and villi height:crypt depth ratio were reduced in poults fed diets containing CFP. Given the changes in lipid concentrations of HP-DDG versus CFP (HP-DDG at 8.89% compared to CFP at 7.57%; Table 3), it is plausible the ileal villi height:crypt depth ratio difference may be reflective of lipid concentrations in the diet versus the tested products.

In conclusion, DDGS, HP-DDG, and CFP are excellent sources of AME_n_ and digestible AA in turkey diets. Diets containing up to 15% HP-DDG or CFP can be fed to turkey poults without affecting overall growth performance, intestinal health, or mortality.

## DISCLOSURES

The authors declare that a portion of this manuscript was funded by The Anderson Inc. who donated the HP-DDG and CFP used in Experiment 2 in this manuscript and provided financial assistance to support a portion of manuscript. The financial support supported the first authors in their graduate program, farm costs associated with the trial, and assay costs. This declaration is provided in the manuscript under the disclosure section.

Mention of a trade name, proprietary product, or specific equipment does not constitute a guarantee or warranty by the USDA, University of Minnesota, or Iowa State University and does not imply approval to the exclusion of other products that may be suitable. The USDA is an equal opportunity provider and employer.
